# *QuickStats*: Percentage* of Adults Aged ≥18 Years Who Have Been Bothered a Lot by Headache or Migraine in the Past 3 Months,^†^ by Sex and Age Group — National Health Interview Survey, 2021^§^

**DOI:** 10.15585/mmwr.7222a6

**Published:** 2023-06-02

**Figure:**
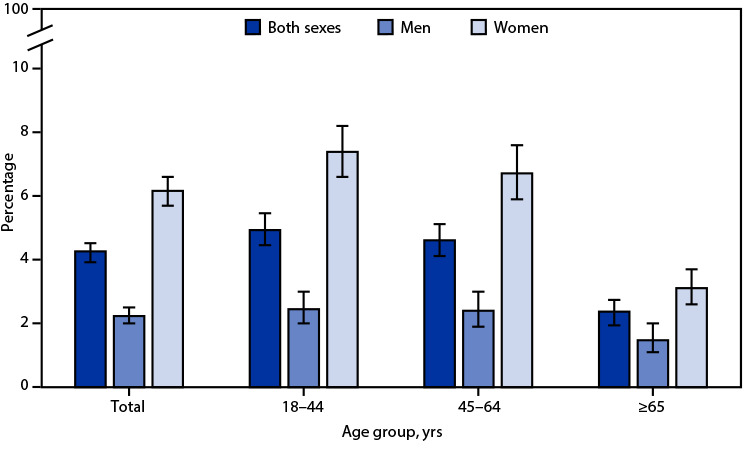
In 2021, 4.3% of adults aged ≥18 years reported being bothered a lot by headache or migraine in the past 3 months with the percentage among women (6.2%) higher than that among men (2.2%). Percentages were higher among women than men in all age groups: 7.4% versus 2.5% in adults aged 18–44 years, 6.7% versus 2.4% in those aged 45–64 years, and 3.1% versus 1.5% in those aged ≥65 years. Among men and women, the percentage of those bothered a lot by headache or migraine in the past 3 months was lowest among those aged ≥65 years.

**Source**: National Center for Health Statistics, National Health Interview Survey, 2021. https://www.cdc.gov/nchs/nhis.htm

